# Has the new GP contract in Scotland reduced health inequalities? Qualitative evaluation of the views of general practitioners working in deprived areas

**DOI:** 10.1186/s12939-025-02609-w

**Published:** 2025-09-26

**Authors:** Laura Aitken, Eddie Donaghy, Stewart W. Mercer

**Affiliations:** Usher Institute, College of Medicine and Veterinary Medicine, University of Edinburgh, Old Medical School, Teviot Place, Edinburgh, EH8 9AG UK

**Keywords:** Health inequalities, General practice, Primary care, Scotland, Contract, Reforms.

## Abstract

**Background:**

Scotland has the widest health inequalities in western Europe and a well-documented inverse care law in general practice. Scotland introduced a new General Practitioner contract in 2018, reforming how care is delivered. Changes included expanding the primary care multidisciplinary team, and grouping practices into geographical clusters to improve quality of care for the local populations. A stated aim of the new contract was also to reduce inequalities in health. However, the effects of the reforms upon health inequalities have been little explored. This study aimed to analyse the views of General Practitioners working in deprived areas on the impact of the contract on health inequalities in Scotland.

**Methods:**

This study involved a secondary analysis of qualitative data from one-to-one interviews with 11 GPs serving patients in deprived areas of Scotland. Thematic analysis was used to analyse the data.

**Results:**

Despite some positive opinions on some aspects of the contract, GPs in deprived areas felt that the aim of reducing inequalities had not been achieved. Reasons for this were: (1) persisting barriers to engagement for patients in deprived areas (including poor access to services, lack of patient education about the reforms, centralisation of some services, and difficulties with remote consulting), (2) inadequate support to manage patients with complex problems (including difficulty in providing continuity of care, and limited resources for patients with specific comorbidities such as mental health and chronic pain), (3) clusters in areas of deprivation lacking capacity to address health inequalities (including lack of time, lack of training, and lack of data and evaluation), and (4) a lack of workforce and strategic planning in the new contract regarding deprivation (such as suitable resource allocation and recruitment of sufficient numbers of appropriate staff in practices in deprived areas). Two additional cross-cutting themes were identified, relating to lack of time and poor relationships.

**Conclusions:**

The new Scottish General Practice contract has not achieved its aim of reducing health inequalities, according to General Practitioners working in deprived areas. Future iterations of the contract need to implement changes that will tackle the inverse care law and thus help reduce inequalities in health.

## Background

Health inequalities are the presence of unfair, avoidable differences in health, and are a global problem, both between and within countries worldwide [1]. Scotland has both ‘the widest socioeconomic inequalities in health and the worst overall population health in Western Europe’ [2]. Key underlying drivers for persisting and widening health inequalities in Scotland include accumulation of severe multiple disadvantages, a lack of improvement in living standards, and the effects of austerity on healthcare services [3]. As regards healthcare, the National Health Service (NHS) in the UK – along with many other countries in the world [4] has long suffered from the ‘inverse care law’, by which those in greatest need of healthcare are the least likely to receive it [5]. The inverse care law was described in general practice in Scotland almost twenty years ago with patients in deprived areas having greater healthcare need, yet receiving shorter and less enabling consultations than patients living in more affluent areas [6]. This mismatch of need and supply has been related to underfunding of general practices in deprived areas relative to patients’ health status [7] and most affects patients with complex problems such as multimorbidity [8].

One important way in which health inequalities may be addressed is thus through the delivery of equitable primary care services, and in April 2018 a new General Medical Services (GMS) contract was implemented to deliver radical changes to general practice in Scotland, with an aim of transforming how care is delivered and a stated aim of reducing health inequalities [9]. Key changes included the abandonments of the quality and outcome framework (QOF), the formation of GP clusters – groups of practices working together to improve the quality of care based on local population needs, and the expansion of the primary care multidisciplinary teams (MDT) to help reduce GP workload and allow GPs to spend more time with those patients with complex problems such as multimorbidity. Evaluation of the new contract overall has found that progress has been slow, clusters have insufficient support, and despite a rapid expansion of the MDT, many problems exist with their effectiveness and integration into GP practices [10, 11]. These previous publications did not, however, fully explore the views of GPs working in deprived areas on whether health inequalities have been reduced by the new contract.

The aim of this study was to conduct a secondary analysis of our previous qualitative interviews to explore the views of GPs who work in deprived areas on the impact of the new general practice contract on health inequalities in Scotland. Specifically, we wanted to explore GPs views on if and how expansion of MDTs and cluster working have impacted upon inequalities.

## Methods

### Study design

This study utilised data which had previously been collected as part of a wider mixed methods evaluation of the Scottish primary care reforms [10–13]. A secondary analysis was conducted of qualitative data, which had been collected through semi-structured telephone interviews with GPs between 2021 and 2022.

### Sampling and recruitment

This study was based upon qualitative data, collected in the form of semi-structured interviews with 11 GPs working in in urban deprived areas, as previously described [10]. Practice deprivation level was estimated from the deprivation level of all patients registered with the practice, derived from the patients’ postcodes using the Scottish Index of Multiple Deprivation (SIMD) [14]. In Scotland, patients must register with one practice only. Those who live in the 15% most deprived areas (as determined by SIMD) are considered high deprivation by the Scottish Government [14]. All practices in Scotland can then be ranked based on the percentage of registered patients living in the 15% most deprived areas [15]. The 11 GPs interviewed all worked in practices ranked in the top quintile of deprivation (nine from practices in the most deprived decile and two from practices in the second most deprived decile).

### Data generation and processing

Data were collected through one-to-one, semi-structured interviews, conducted in two waves, firstly from March and May 2021 [10] and then from May to June 2022 [11], exploring a range of aspects of GP viewpoints on the implementation and effects of the primary care reforms. The first wave of interviews included seven GPs and the second wave had 4 GPs. Details of the GPs interviewed are shown in Table 1 (we are limited in how much information we can include due to the need to preserve confidentiality). Specific questions were asked regarding whether the GPs felt the reforms were reducing health inequalities. The original analysis in the two previously published papers [10, 11] only briefly touched on the issue of deprivation and health inequalities, and thus a deeper exploration was warranted in the current analysis.

**Table 1:** Characteristics of participating gps

GP number	GP gender	Years qualified as a GP	Years in current practice
**1**	Female	21	10
**2**	Female	34	28
**3**	Female	30	25
**4**	Female	25	25
**5**	Female	29	22
**6**	Male	31	31
**7**	Male	17	15
**8**	Female	9	5
**9**	Male	8	7
**10**	Male	11	8
**11**	Male	16	2

### Data analysis

The interviews, which had been transcribed verbatim, were analysed using reflexive thematic analysis [16] using NVivo (version 14). One interview was coded by all three researchers, and another by two of the researchers (ED and LA), and a coding frame agreed. LA then coded the remaining nine interviews. Codes were organised into themes through an iterative process involving regular meetings and discussions between all three researchers. We followed the Standards for Reporting Qualitative Research Framework [17] and good practice principles [18].

### Ethical approval

Ethical approval was in place for the overall evaluation (REC reference: 21/WA/0078), which included approval for this study. Further ethical approval for LA’s involvement was gained from the Usher Masters Research Ethics Group (UMREG), Usher Institute, University of Edinburgh (UMREG ID: 24320).

## Results

Overall, analysis of data from the GP interviews revealed a general agreement that primary care reforms in Scotland have not achieved their stated goal of reducing health inequalities. Four key themes were identified, alongside two cross-cutting themes.

### Theme one: barriers remain to patient engagement in deprived areas

#### Difficulties remain for some patients in deprived areas in accessing care

Several interviewees also discussed that often patients with the greatest need may not be those who are most likely to seek care.

*‘patients who don’t demand a lot*,* but who probably should see me*,* are not getting to.’* (GP 10).

Some GPs raised concerns that the practical barriers to access remain for many patients in deprived areas, such as transportation and difficulties in engaging with appointment systems.

*‘if you are living in a deprived area and you don’t have a car*,* you’re frequently changing address or you’re sofa surfing….there are so many barriers to people being able to make use of that service that it’s completely unfit for purpose for a lot of people who really need it’* (GP 2).

#### Better engagement with patients in deprived areas was felt to be important in bringing them ‘on board’ with new ways of working

Many GPs felt that the reforms to primary care had not sufficiently involved and engaged the public, which was felt to be a particular issue in deprivation.

*‘You absolutely have to take the public with you otherwise they feel that things are being taken away from them*,* and that’s really difficult.’* (GP 2).

#### There have unintended consequences of centralising some services, creating additional barriers for some patients

GPs reported that services which had been centralised as part of the reforms made access more difficult and potentially excluding some patients in deprived areas.

*‘if you take immunisations out of general practice*,* particularly in deprived areas*,* then you’re effectively creating a barrier to access to immunisation which will be more difficult for patients in deprived areas to overcome.’* (GP 6).

It was also noted that, previously, such appointments often allowed for an opportunistic medical reviews and some of this valuable continuity had been lost.

#### Increased use of remote consulting presents a further barrier to accessing care for patients in deprived areas

‘Digital poverty’ including ack of access to telephone and internet services was felt to have resulted in difficulty for many patients in deprived areas in terms of contacting practices, making appointments, and participating in remote consultations. The onset of the Covid-19 pandemic exacerbated this, when remote consulting was more widely adopted.

*‘it’s not just elderly people. It’s*,* you know*,* lots of people in their 30 s and 40 s are digital impoverished.’* (GP 4).

‘*so much of it depended on the other person’s connection*,* not ours. And also their ability to use it*,* their education level to use it and their access to it. And that’s a huge health inequality’* (GP 11).

### Theme two: the contract has not addressed the challenges of managing patient complexity in deprived areas

#### Continuity of care is of key importance in areas of deprivation and in managing complexity, but this is often not possible to deliver, and development of MDTs May have resulted in more fragmented, less holistic care for patients in deprived areas

It was emphasised by most GPs that patients in deprived areas commonly have complex healthcare needs, and such patient particularly benefited from continuity of care.

*‘I’ve been here for 25 years*,* if I’ve known somebody for 25 years and they’ve got mental health problems*,* they would just feel*,* I hope*,* very confident talking to me*,* I know them really well*,* I know everything that’s happened to them for 25 years*,* they know me.’* (GP 1).

However, it was widely felt that the new contract had not improved continuity and felt that the extension of the MDT had negative effects on continuity of care, resulting in more fragmented care.

*‘I think there are isolated incidents where physios or pharmacists can manage quite simple*,* well-defined pieces of work*,* but the way the job is now is that we are dealing with huge amounts of complex cases which we are the only people who can really manage that.’* (GP 10).

#### High levels of poor mental health, chronic pain and stressful life experiences in areas of deprivation are impossible to manage effectively within the constraints of the current system

Several GPs highlighted the difficulties in managing patients with complex needs such as chronic pain and mental ill health, which are often part of multimorbidity and compounded by stressful life circumstances in deprived areas.

*‘my area desperately needs a pain service or a really good mental health service…We haven’t got any way of trying to make a service happen’* (GP 7).

*‘I think*,* in those populations*,* mental health has a massive effect on the*,* a massive impact on the effect of multimorbidity. I think it makes multimorbidity feel worse*,* and outcomes worse. So I actually think that having mental health support makes a difference to that.’* (GP 1).

### Theme three: clusters lack capacity to tackle health inequalities

#### GPs felt there has been a lack of support and training around cluster working in deprived areas

Most GPs interviewed described benefits of the intrinsic cluster function, where practices were coming together to collaborate, share ideas and work on quality improvement projects. However, they felt that training and support was inadequate, and their ability to participate in quality improvement projects was limited by time and workload pressures day to day.

*‘I think the clusters have been variable*,* but I think a lot of them have struggled with having enough support*,* and time*,* and training’* (GP 1).

*‘We are not getting the support. I think in general people would say that we are not getting the support that they could*,* I think that’s what other CQL’s would be saying*,* that I have not been getting very much’* (GP 5).

#### The extrinsic function of clusters has not been fully realised

Interviewees also felt that the external role of clusters was not be achieving its aims.

‘*the extrinsic functions of the cluster are*,* in general*,* poorly developed*,* which is influencing wider system change*,* reaching out into secondary care and social care and influencing how local systems develop and improve*,* very poorly developed’* (GP 2).

#### There has not been enough use of data or evaluation regarding health inequalities

It was widely felt that there was a lack of evaluation to assess what had or had not worked well, and to allow this to inform planning decisions.

(referring to changes set out in the reforms) *‘I think they should be reviewing them and saying like*,* which ones are going to be of best benefit and the ones that are going to be of most benefit….are the ones that we should be putting the money into. The ones that aren’t of any benefit*,* the money shouldn’t be going into that’.’* (GP 5).

#### There has been inadequate focus on the wider determinants of health

Alongside specific issues with cluster working, interviewees felt that the reforms had not placed enough emphasis on the wider determinants of health.

*‘a lot of chronic disease management would be helped by things outwith medicine*,* to do with*,* you know*,* access to green space*,* access to supported exercise*,* access to healthy food*,* all those*,* and how to cook it*,* and all those sorts of things….there’s a much wider setting there that hasn’t been addressed*,* or some has been addressed patchily’* (GP 1).

### Theme four: issues around workforce and strategic planning have limited how effective the reforms have been in reducing health inequalities

#### MDT working has not released additional time for gps to manage complex patients

Aside from the issue of fragmentation of care (as discussed in sub-theme 2.1), other problems with MDT staff were raised such as the mismatch between what practices needed and what they received.

*‘the reality is that the workforce is not there to extend the team. There aren’t the pharmacists*,* there aren’t battalions of pharmacists sitting at the top of the hill waiting to charge down and join general practice. There aren’t physiotherapists*,* there aren’t advanced nurse practitioners.’* (GP 2).

Alongside this, GPs were required to train and mentor new staff members along with their other usual duties.

*‘we get ANPs who are in training rather than trained. So at the moment*,* whenever you get an ANP coming on to your team*,* you’re expected to train and mentor as well. So actually your workload’s increasing for two…for the first two to three years.’* (GP 4).

#### There has been a lack of workforce planning to address the inverse care law, and challenges remain with recruitment and retention of staff in areas of deprivation

GPs felt that there had been inadequate attention to developing the workforce in areas of deprivation, especially in early careers (both doctors and allied health professionals). Attracting staff who had an interest in working in deprived areas was important, with the right mix of MDT staff.

*‘recruitment and retention is difficult in this area*,* I think because of where we are and probably we need people to live local and work local. I think in that respect we maybe don’t have the full availability of things that we could have to help us.’* (GP 8).

*‘we’ve had nurse practitioners come to us who had no desire to work in a deep end practice*,* and that is really difficult because it’s not going to work for them. It is a deep end practice*,* it’s got a very specific flavour*,* and you either want to work there or you don’t. You know?’* (GP 2).

#### Strategic planning and resource allocation have not adequately addressed inequalities in health

Further to the issue of workforce planning, it was also felt that there had not been suitable consideration of resource allocation in practices in deprived areas through the Scottish Resource Allocation Formula. Several GPs discussed felt that resources had not been suitably weighted towards areas of deprivation.

*‘I don’t think is weighted in the least toward deprived patients.’* (GP 4).

### Cross-cutting themes

#### Lack of time to Meet the aims of the reforms

Throughout the interviews the recurring theme of inadequate time was apparent. GPs described how high workloads meant that they had very little time in their day to devote to anything beyond the immediate management of patients. Lack of time impeded the ability of GPs to spend more time on the management of patients with complex problems.

*‘the new contract has to be implemented with all the staff that they promised us so I can work at that level. But my days are spent firefighting and looking after sick people. And that never…that work never ends’* (GP 4).

Time was also a limiting factor in the ability of many GPs to engage fully in cluster working.

*‘most GPs haven’t wanted to be a cluster lead because although there’s a payment for it*,* there’s a lot of…it’s all about time and extra time.’* (GP 3).

#### An inadequate focus on Building relationships to tackle health inequalities in deprived areas

Relationships was a common theme, from the individual relationships patients have with their doctor, to wider relationships in the primary care team, and other interfaces (such as social work). Many interviewees felt the reforms had not placed a high enough priority on relationship building.

*‘The managers and the Health and Social Care Partnership*,* the integration is all very management type things and it is really alien to most GPs. So difficult to get involved in the decision making higher up.’* (GP 7).

It was also suggested that bringing together primary care and public health in a more cohesive way would be beneficial, and that building relationships with public health colleagues would be desirable.

*‘if we think of public health as the macro population health partner and primary care as the micro population health partner*,* I think that we need to see much more investment and collaborative working between the two. Whether it’s through dual training*,* whether it’s through sense checking each other’s policy development*,* primary care inputting into national strategy. Public Health Scotland helping clusters and practices more. I just think there’s so much more scope for collaborative working in terms of improving population health and it would be great to see that included.’* (GP 2).

## Discussion

### Summary of findings

Overall, analysis of data from the GP interviews revealed a general agreement that primary care reforms in Scotland have not achieved their stated goal of reducing health inequalities. Thematic analysis of the interview data revealed four key themes: (1) barriers remain to patient engagement in deprived areas; (2) the contract has not fully addressed the challenges of managing patient complexity in deprived areas; (3) clusters lack capacity to help reduce health inequalities; (4) issues around workforce and strategic planning have limited how effective the reforms have been in reducing health inequalities. Each of these themes had sub-themes, which are further discussed above. Alongside these four key themes, two further cross-cutting themes were identified: (1) lack of time to meet the aims of the reforms, and (2) an inadequate focus on building relationships to tackle health inequalities in deprived areas.

### Relationship to published literature

The results of this study can be seen in context with evaluation of primary care reforms in Scotland to date. In 2019, the Scottish Government published a ‘national monitoring and evaluation strategy for primary care in Scotland’ [19], to assess the impacts of the reforms in primary care, extending until 2028. Results of survey data thus far have shown that despite some benefits, MDTs were not yet achieving their intended aims. There was variability in MDT provision and limitations to how much this had improved GP workload [20], and mixed views overall as to whether MDT members felt their role had impacted upon health inequalities [21]. GP respondents suggested overall that in areas of higher deprivation, access to urgent care staff and mental health practitioners was lower overall [20].

Recent research in Scotland has also shown that the inverse care law persists, with more deprived areas having lower numbers of whole-time equivalent (WTE) GPs than affluent areas despite a two to three-fold gradient in need (poor health) of patients between affluent and deprived areas [22]. A report funded by the Health Foundation on the inverse care law in Scotland concluded that there is an ‘implementation gap’ between the aims of reducing inequalities as stated in the new contract and what has been achieved in practice [12]. Quantitative analysis demonstrated that payments to practices across deciles of deprivation do not reflect the greater level of need in more deprived areas, and further data suggests that it is unclear whether multidisciplinary team expansion has been ‘adequately distributed according to local population need’ [12]. In other work, there were also notable differences between patient experiences of GP consultations, with poorer experiences in deprived urban areas, which included measures of overall satisfaction with the consultation, perceived GP empathy, enablement, and improvement of symptoms [13].

The results of this study can also be taken in context with research more broadly in the United Kingdom and beyond. Reforms such as the introduction of Primary Care Networks (PCNs) in England has not been shown to have significantly improved longstanding inequalities in workforce distribution [23]. Increased demand upon general practice, linked with reduced continuity of care and difficulty for patients in navigating complex systems, have also been shown to exacerbate health inequalities in the north of England [24]. Providing further context to the reforms from an international perspective, a systematic scoping review, published last year, examined evidence around primary care transformation in OECD countries and China [25], and results suggested that internationally, there have been a wide range of approaches to primary care transformation, with variable and often limited data on the impact of these upon health inequalities. Several barriers and facilitators to successful implementation of reforms were also noted, including the importance of good relationships in primary care as a key facilitator, and lack of time being a barrier [25].

### Strengths and limitations

One particular strength of this project was that it uses direct, in-depth data collected from GPs working in frontline primary care services in Scotland in deprived areas. Having a range of GPs from different health boards and areas of deprivation was helpful in allowing a spectrum of views to be explored, as was including a mix of frontline GPs, cluster leads, and senior national stakeholders who were also practising GPs in deprived areas. There was a good mix of male and female GPs, and a range of years qualified in general practice, and working in their current practice. More than half had been in their current practice for 10 years or more, and thus had experience of working in a deprived area before the new GP contract started. In common with other qualitative research, the results of this project could not necessarily be said to be generalisable or transferable, however the results are compatible with recent quantitative findings on the continuing inverse care law in general practice in Scotland [22, 26].

Furthermore, the interviews used were completed between 2021 and 2022, and so there may now be differences in what is happening day to day in practice. However, we believe that our findings remain pertinent for several reasons. Firstly, the second phase of the new GP contract in Scotland remains under negotiation, and there have been no other major national policy changes regarding deprivation and general practice since the 2018 contract (phase 1). Secondly, our findings are supported by more recent work funded by the Health Foundation on the new GP contract and the inverse care law in deprived areas of Scotland [12] and by our own recent quantitative evaluation from national surveys of GPs views [27]. Furthermore, Audit Scotland (the independent watchdog of public services in Scotland) recently published their own evaluation of the new GP contract in Scotland and came to similar conclusions as ourselves [28]. It also may be the case that’s some of the issues highlighted may have worsened, particularly in relation to the wider sociopolitical climate [29].

An additional possible limitation was that the interviews were conducted by telephone. Whilst this may have some potential advantages, it may also have impacted upon the results, as whilst there is evidence to suggest that telephone interviewing is effective and is a well-accepted method for obtaining qualitative data, in-person interviews can potentially provide additional depth of responses, and improve rapport between interviewer and interviewee [30].

There are also other forms of health inequalities which this data did not explore, and this may be a further limitation in understanding the impact of reforms on health inequalities overall in Scotland. This may include inequalities in health due to gender, race, ethnicity, or disability [31–33].

### Implications for future practice

The results of this study support recommendations from the Royal College of General Practitioners and from the aforementioned Health Foundation report, which suggest key improvements to primary care are needed including increased funding, proportionate to level of need, greater support for clusters [12], stabilisation of the primary care workforce, and a need for longer consultations (particularly in deprived areas). The results of this study also emphasised the importance of continuity of care, which also supports the findings of the Health Foundation report.

Of course, health inequalities are not only due to access to health services, but are related to inequalities in general and the wider determinants of health. Long-term solution will therefore require structural changes in the social determinants of health such as employment, income, education, housing, and so on. However, healthcare itself is also a social determinant and if NHS healthcare is not at its best where it is needed most, the continuation of the inverse care law will simply contribute to further inequalities in health. As negotiations for phase two of the Scottish GP contract continue (at the time of writing), a number of promising pilot studies have/are being conducted by the Scottish Government, including four demonstrator sites to more fully implement Pharmacotherapy and Community Treatment and Care (CTAC) services in the multidisciplinary teams [34], longer consultations for patients with complex needs in deprived areas [35], an enhanced service for early detection of cardiovascular disease in general practice [36], and resources to enhance family wellbeing through the Whole Family Wellbeing Fund [37].

## Conclusions

The new Scottish General Practice contract has not achieved its aim of reducing health inequalities, according to General Practitioners working in deprived areas. These include barriers to engagement for patients in deprived areas, inadequate support to manage complexity in deprived areas, clusters lacking capacity to address health inequalities in deprived areas, and issues around workforce and strategic planning. Along with these, issues around lack of time and relationships were also reported. Future iterations of the contract need to implement changes that will fundamentally tackle the inverse care law and thus help reduce inequalities in health in Scotland.

## Data Availability

No datasets were generated or analysed during the current study.

## Abbreviations

GMS: General Medical Services
GP: General Practice or General Practitioner
HI: Health Inequalities
MDT: Multidisciplinary Team
NHS: National Health Service
PCN: Primary care Network
SIMD: Scottish Index of Multiple Deprivation
WTE: Whole-Time Equivalent
UMREG: Usher Masters Research Ethics Group
